# Reducing harm through the development of good preparation practices for the injection of slow release morphine sulphate capsules

**DOI:** 10.1186/s12954-020-00389-w

**Published:** 2020-07-16

**Authors:** Lenneke Keijzer

**Affiliations:** Research and Prevention Fund Apothicom “know more, risk less”, 52 Avenue Edison, 75013 Paris, France

**Keywords:** Filtration, Injecting drug use, Harm reduction, Morphine sulphate, Sterifilt, Wheel filter, Best preparation practice, Non-medical use of prescription drugs

## Abstract

**Background:**

It is not always easy to advise people who inject drugs (PWID) on how to prepare their drugs in a way that is associated with reduced harm. This is particularly true for pharmaceutical drugs that are not meant to be injected. Our objective was to find “good preparation practices” for slow release morphine sulphate capsules, namely preparation methods that reduce harm, that are evidence-based and acceptable to PWID.

**Methods:**

In the laboratory, morphine sulphate capsules were prepared using both a cold and lukewarm preparation technique, two contact and stirring durations (1 min and 20 s) and 4 different filters (cotton filter, Sterifilt, Sterifilt+ and a wheel filter). The following outcomes were compared: particle reduction and morphine content in the filtrate, as well as filtration ease and time.

**Results:**

The lukewarm method and a stirring and contact time of 1 min were associated with a considerably higher morphine yield than both the cold method and the stirring time of only 20 s. Moreover, the suspension obtained was easy to filter using membrane filters. Particle reduction was important with all three membrane filters tested. Using the lukewarm method, morphine recovery was 86% for the wheel filter, 89% for the Sterifilt and 99% for the Sterifilt+.

**Conclusions:**

The provision of a method that is easy to use, reduces harms associated to the injection of insoluble particles and recovers virtually all the active drug has a large chance to be adopted by people who use drugs. This type of “best practices” can be provided by drug workers and by people who use drugs to actively promote harm reduction.

## Objective

This study was set up to examine the influence of different preparation practices, including filtration, on particle count, morphine concentration and filtration ease. Our objective was to find “good preparation practices” for slow release morphine sulphate capsules, namely preparation methods that reduce harm, that are evidence-based and that are acceptable to people who inject drugs (PWID). Ideally, this type of practices will result in the elimination of harmful insoluble particles associated with an easily filterable solution, and a high morphine content, encouraging people who inject drugs to use this method.

## Background

Besides illicit drugs such as heroin and cocaine, some PWID inject pharmaceutical drugs. The injection of such drugs is becoming more and more common in many countries, like France, the USA, Canada and Australia [1–7].

There may be different reasons for using or even preferring pharmaceutical drugs. They may be easier to obtain in some contexts, their nature and dosage are known, and their use may be more acceptable in some groups.

The injection of pharmaceuticals entails complications which, for some, are inherent to insoluble tablet fillers such as talc, starch and microcrystalline cellulose. Their injection can give rise to several complications such as abscesses, ulcers, granulomas and phlebitis, pulmonary embolism, fibrosis and talcosis [8–11]. Filtration can prevent or delay these complications by removing insoluble particles from the suspension prior to injection. Membrane filters, such as the Sterifilt and wheel filters, are more efficient in eliminating particles from a solution than cotton or cigarette filters that are commonly used by PWID [12–14].

In France, one of the pharmaceuticals commonly diverted by injection is Skenan, a slow release capsule formula of morphine sulphate. The injection of this drug increased from 7 to 17% among people attending needle exchange programmes and drop-in centres between 2003 and 2015 [15, 16].

The major preparation method for this drug involves heating after adding the active substance to the solution [17, 18]. Because of the presence of starch, when heated, the suspension becomes highly viscous. Therefore, the more efficient membrane filters tend to clog up and are not often used. Keijzer and Imbert [18] found that only 11% of the people who inject this drug used membrane filters, while these have been accepted and are now widely used for the filtration of drugs such as buprenorphine that are extracted from standard, non-slow release tablets.

New drug preparation guidelines have also been taken up by PWID in other contexts [19]. But, despite the promotion, by harm reduction professionals of a cold preparation technique for Skenan, this advice has not been followed up [17]. It is thus plausible that there is a good reason to heat this drug. Some mention a lack of tingling feelings when they use the cold technique and a membrane filter; others mention an enhanced high when heating [20].

This might be true. The solubility of morphine is 53.8 mg/mL at a temperature of 25 °C [21]. Most PWID in France inject 100-mg or 200-mg capsules; they may heat in order to dissolve the available morphine in a small volume (1-mL and 2-mL syringes are used). McLean et al. [22] showed that heating does not increase the solubility of morphine sulphate. However, they used a volume of 3 mL to dissolve 60 mg of morphine sulphate, and they waited 5 min before filtering the suspension. Low solubility may not be a limiting factor under these conditions. According to other sources [23, 24], a higher temperature does seem to enhance morphine or morphine sulphate solubility.

Our hypothesis is that more morphine is extracted in a 2-mL solution after heating.

### Preparation practices used in the field

The hot preparation method, commonly used for slow release capsules of morphine sulphate (81% of the users of this drug heat their solution -[20]), is performed as follows: The microbeads contained in the capsule are placed in a cooker (either whole, or after being crushed), water is added and the solution is subsequently heated until the first bubbles appear. Thereafter, the suspension is stirred and filtered through either a small piece of cigarette filter or a cotton filter.

Though the majority use this method, other preparation methods are used.

Though very rare (personal observation in the field), some people use the cold preparation method. They crush the microbeads, put them in the cooker, add water, stir, wait for about 5 min, stir again and filter. Membrane filters can be easily used with this preparation method.

Another method has been developed by the attendees of a harm reduction centre in France that is managed by a drug users’ organisation (ASUD, Nîmes). This technique seems promising as it is used by a large number of users, and it is compatible with membrane filtration. We will call this “the lukewarm method”. First of all, the microbeads contained in the capsule are crushed and put aside. Water is poured into the cooker and heated until the first bubbles appear. Fairly quickly, the crushed powder is added to the lukewarm water and the solution is stirred. Subsequently, the solution is filtered either through a membrane filter only or through a membrane filter that is positioned on top of a cotton filter, called combined filtration. Prepared in this fashion, the solution is still a little viscous, but it goes through membrane filters; the presence of a cotton filter underneath prevents the filter from clogging and enhances the filtration speed.

Many professionals in the field of harm reduction (including the author) have been wondering for a long time what would be the best technique for the preparation of this drug. The manufacturer of Skenan® turned to the Research and Prevention Fund Apothicom receiving a question from a drug users’ organisation (Psychoactif) on the harms associated to the injection of Skenan. This pushed the fund to write a protocol and to subsequently request Ethypharm to perform the lab work on its product.

Ethypharm Laboratories has been marketing Skenan® and Actiskenan® specialties since July 2015. Skenan®, mainly in the 100-mg and 200-mg dosage strengths, as well as other Morphine specialties, have been used off-label as an opiate substitution drug since the early 1990s. This “ancient” use is well documented (OFDT—French monitoring centre for drugs and drug addiction; Oppidum—monitoring centre for psychotropic drug that are illicit or diverted from their pharmaceutical use; Opema—monitoring centre for drug dependencies in ambulatory medicine). This use is not framed by a legal regulation, except the “Letter Girard” of 1995 that states that, in the absence of a marketing authorisation as a substitution drug, Skenan can only be prescribed as such in rare cases and after concertation between the general practitioner and the medical advisor of the social security. The lack of a clear regulation places health professionals and their patients in condition of random treatment success. Morphine as an option of opiate substitution, even for a limited number of patients, deserves to be explored and better supervised for the benefit of all.

This off-label use of morphine, including Skenan®, as an opiate substitution therapy is clearly not promoted and encouraged by Ethypharm.

## Material

The following items were used to prepare and filter the suspensions: Skenan® 100 mg and Skenan® 200 mg (Ethypharm, France); Maxicup®, a sterile, single use cooker developed to prepare drugs for injection (Apothicom, France); and 2 mL Nevershare® syringes (Exchange Supplies, UK).

**Fig. 1:**
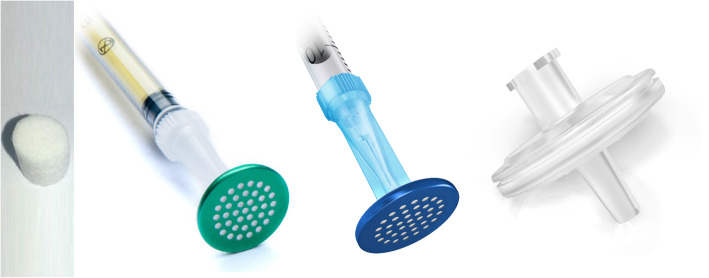
Filters used. From the left to the right: cotton filter, Sterifilt, Sterifilt +, Minisart “wheel filter”

For non-filtered solutions, the suspensions were drawn up through a Terumo Neolus® 25-G needle (Terumo, Japan). For filtration, the following filters were used (Fig. 1):

- Cotton filter, a sterile cotton filter integrated in the Maxicup® blister (Apothicom, France).
- Minisart® PES 0.2 μm, a sterile membrane filter developed for laboratory purposes, compatible with Luer slip syringes only. In the field of harm reduction, they are called "wheel filters" (Sartorius, Germany).
- Sterifilt®, a sterile membrane filter developed for the field of harm reduction, equipped with a 10-μm membrane and compatible with syringes with attached needles and with Luer slip syringes. This is the successor of the Sterifilt described by Scott [14] and Roux et al. [13]. It is characterized by a larger filtration area, increased filtration speed and decreased risk of clogging during filtration (Apothicom, France).
- Sterifilt+®, a sterile membrane filter developed for the field of harm reduction, equipped with a 0.2-μm membrane and compatible with syringes with attached needles and with Luer slip syringes (Apothicom, France).

Other materials used are a hot plate to heat the solutions, standard 80-g white paper and a lighter to crush microbeads and a digital microscope (Keyence VHX 2000) to visualize the insoluble particles in the filtrate.

## Methods

Two preparation practices are thoroughly explored. As the hot method is used most often, it probably results in a reasonable amount of morphine in the solution. However, this method does not resolve any of the complications related to the injection of insoluble particles because the heat renders the solution incompatible with the use of efficient filters. This preparation method has thus not been explored. We decided to focus on the methods that may result in reduced harm, namely the cold method and the lukewarm method.

### Protocol for the cold preparation technique

A 100-mg morphine sulphate capsule was opened, and the microbeads were put on a carefully folded piece of paper. They were crushed using a lighter while inside the paper. The resulting powder was put into the cooker, 2 mL of water was added and the suspension was stirred for 1 min using the rear end of a plunger. The solution was then either directly taken up through a syringe with a 25-G needle or filtered through one of the four filters. As filtration by suction through wheel filters is facilitated by wetting the membrane, these filters were rinsed with 0.3 mL of water prior to filtration.

### Protocol for the lukewarm preparation technique

A 100 mg morphine sulphate capsule was opened, and the microbeads were put on a carefully folded piece of paper. They were crushed using a lighter while inside the paper. The paper containing the powder was put aside for later use. Two millilitres of water was poured into the cooker, which was then put onto a hot plate until the first bubbles appeared. Quickly, the powder was added to the water, and the suspension was stirred for 1 min using the rear end of a plunger. The solution was subsequently either taken up through a syringe with a 25-G needle or filtered through one of the four filters. Prior to filtration, wheel filters were rinsed with 0.3 mL of water.

Three additional tests were performed:

- *Rinsing of wheel filters*: It is known that wheel filters have a high void volume and retain part of the solution [12, 14]. To examine whether more morphine could be extracted by rinsing, one cold and one lukewarm extraction were performed with an additional rinse using 0.3 mL of water after filtration.

- *Contact and stirring time*: All solutions were stirred for 1 min. As 1 min may be long in the context of drug use, a shorter stirring time of 20 s was tested with both preparation techniques and 2 filters, the Sterifilt+ and the wheel filter.

- *Preparation of 200-mg capsules*: Some people use 200-mg Skenan capsules instead of 100 mg. The extraction of 200-mg capsules was tested using the lukewarm method only, the two stirring durations and a Sterifilt+.

The three membrane filters were used alone, a combined filtration was not necessary.

### Data collection

Three tests were performed per preparation technique. The “additional tests” mentioned above have been performed only once. The following results were collected for each sample: total preparation time, total filtration time, volume retention by the used filter, microscopic particle count (digital microscope: Keyence VHX 2000) and morphine concentration. Morphine content refers to the total morphine available in the filtrate, e.g. morphine concentration multiplied with the total volume recovered in the syringe.

To ensure proper particle recognition, dispersions of sole components (soluble and insoluble) in cold and lukewarm water were evaluated. Starch and talc were easily individually recognisable with no significant difference in size and shape of these compounds between cold and lukewarm suspensions.

To measure the morphine concentration, an ICH validated method using high pressure liquid chromatography with fluorescence detection was used.

## Results

### Filtration time

The total preparation time, from opening a capsule to filling a syringe, is about 4 min for the cold method and 5 min for the lukewarm method. Overall, filtration time and ease seem acceptable for all filters. Filtration was faster using the lukewarm method; membrane filters were associated with slower filtration than the cotton filter, and wheel filter filtration was slowest overall.

### Particle retention

**Fig. 2:**
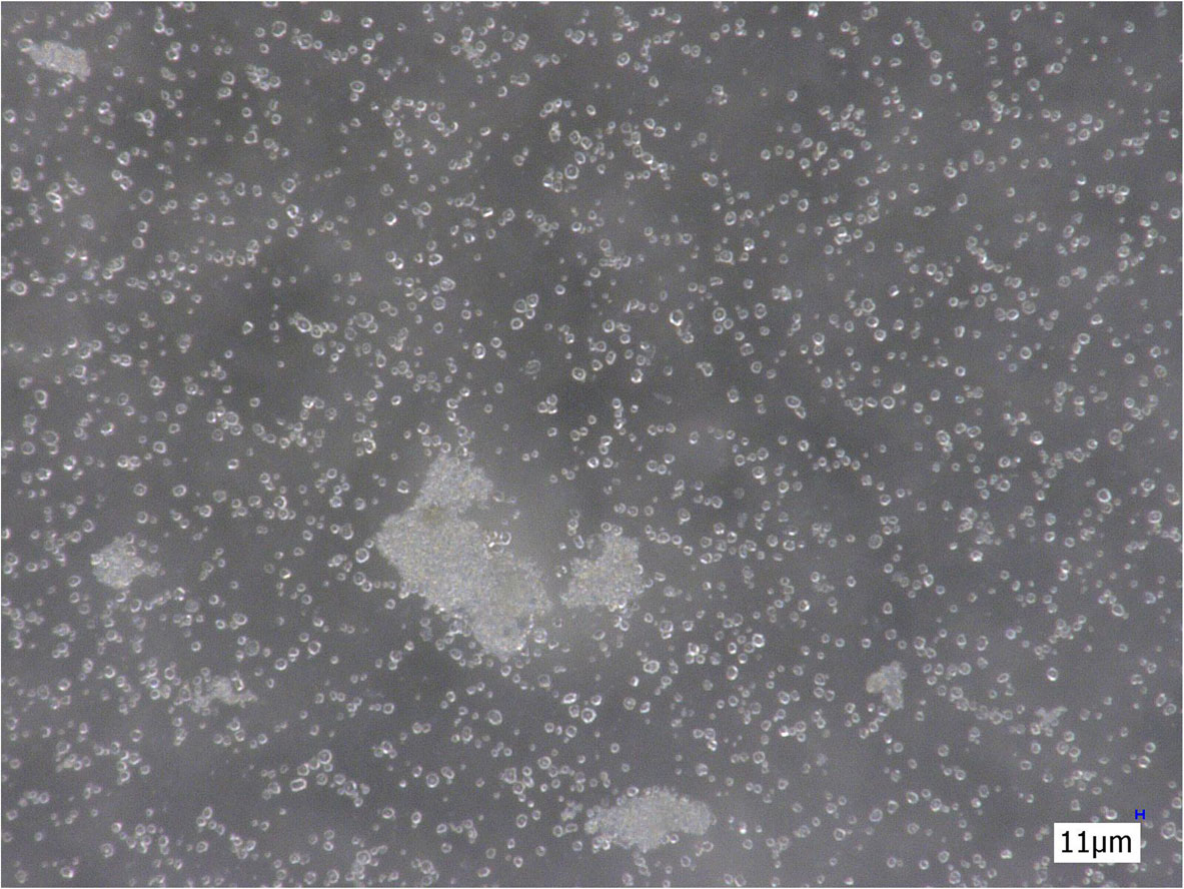
Non-filtered suspension of morphine sulphate (200×)

Without filtration, a large number of insoluble particles are observed in the morphine sulphate solution, consisting of starch and talc particles. The latter are mainly present in clusters (Fig. 2).

**Fig. 3:**
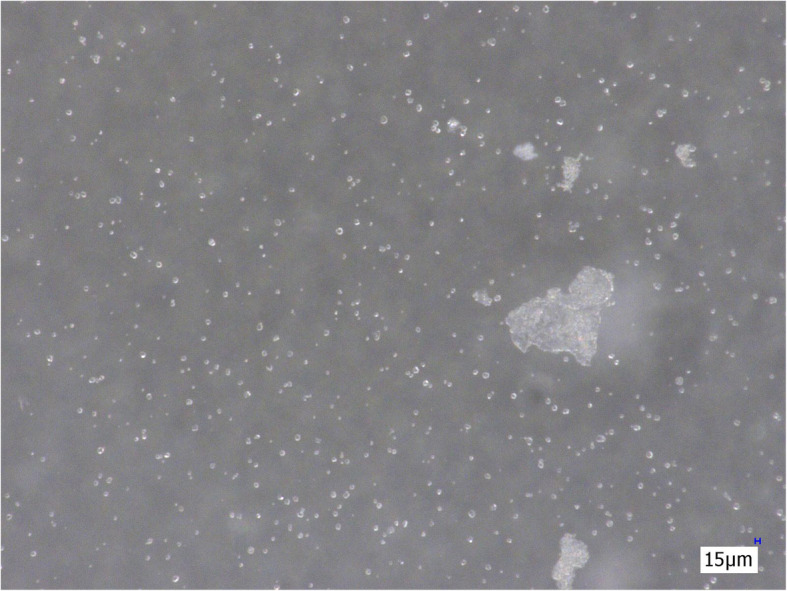
Suspension of morphine sulphate after filtration with a cotton filter (200×)

The cotton filter eliminates mainly starch particles. Though a large number of particles are eliminated by this filter, talc particles are still present in relatively high numbers after filtration (Fig. 3).

**Fig. 4:**
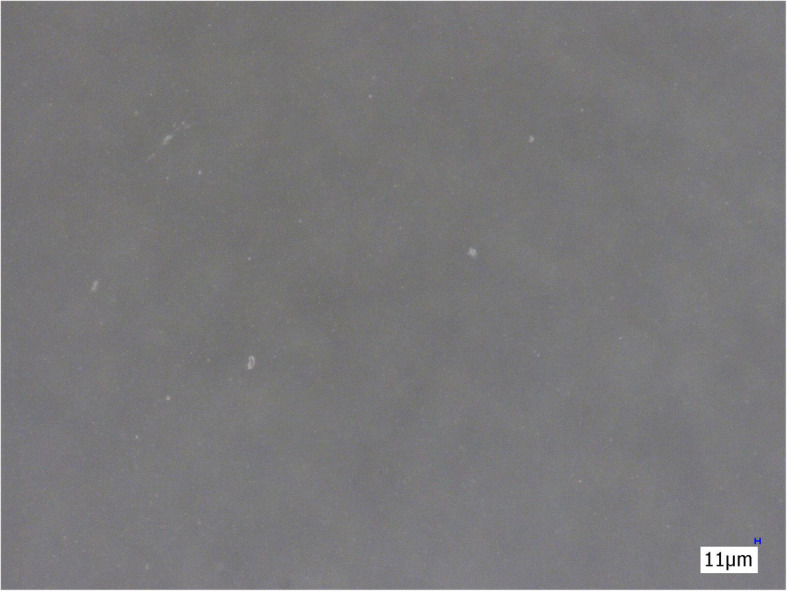
Morphine sulphate solution after filtration with Sterifilt (200×)

**Fig. 5:**
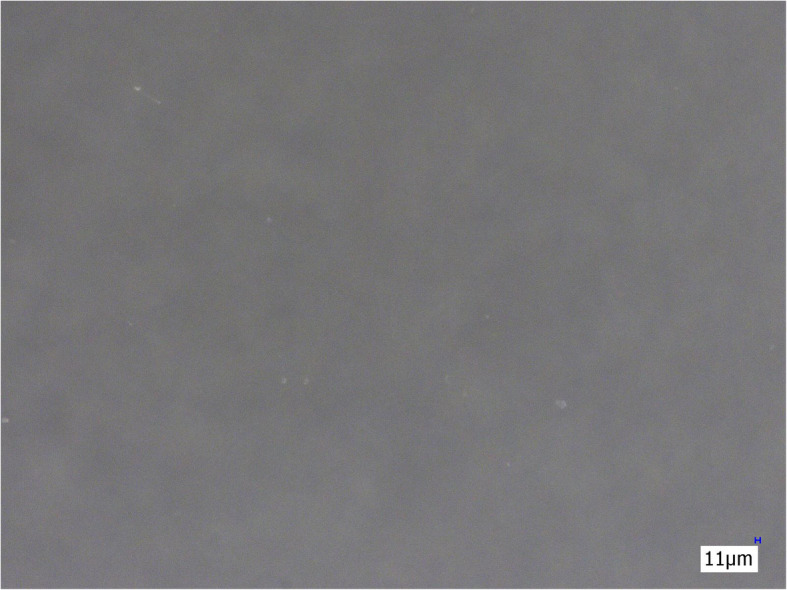
Morphine sulphate solution after filtration with Sterifilt + (200×)

**Fig. 6:**
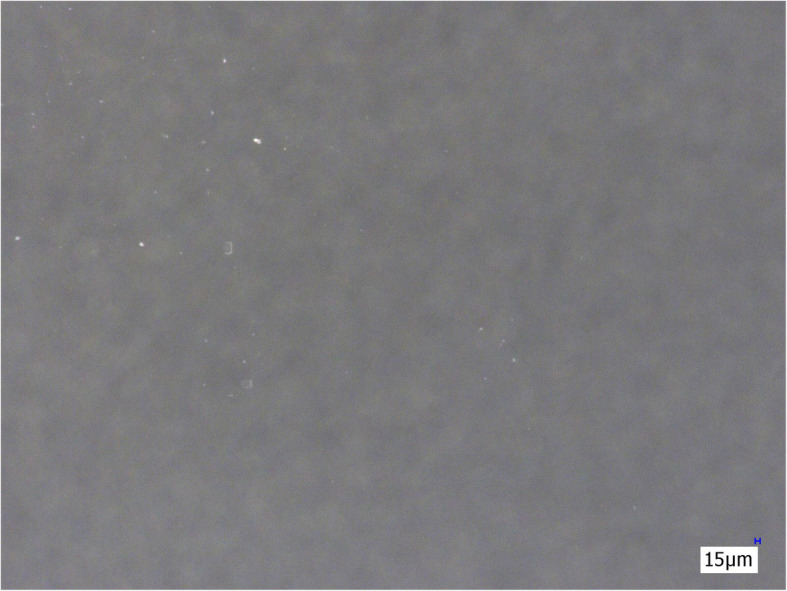
Morphine sulphate solution after filtration with the wheel filter (200×)

The three membrane filters (Sterifilt, Sterifilt+ and wheel filter) eliminate virtually all visible insoluble particles from the morphine sulphate solution (Figs. 4, 5 and 6).

### Morphine extraction

Even though 2 mL of water are used to dissolve 100 mg of morphine sulphate, the cold preparation technique only recovers between 51% (wheel filter) and 65% (Sterifilt) of the drug. Fifty-six percent is recovered without filtration; filtration is thus not necessarily associated to a reduced recovery (Table 1).

**Table 1:** Morphine sulphate extraction from 100-mg capsules using 2 mL of water. Comparison of the cold and lukewarm preparation techniques, according to the filter used

	Mean morphine extraction (%) calculated for the total recovered volume (minimum–maximum)	
	Cold preparation technique	Lukewarm preparation technique
No filtration	56% (50–60%)	97% (96–98%)
Cotton filter	58% (54–62%)	89% (84–93%)
Sterifilt	65% (62–69%)	89% (87–91%)
Sterifilt+	53% (48–58%)	99% (93 to > 100%)
Wheel filter	51% (45–54%)	86% (84–96%)

The lukewarm preparation technique was associated with considerably higher morphine content; extraction was even virtually complete with mean recovery of between 86% (wheel filter) and 99% (Sterifilt+) of the available dose (Table 1). Though the hot method has not been tested, these results show that heating the solution to that point is simply not necessary; all the morphine can be recovered using the lukewarm method.

### Filter effect

The final morphine content differs to some extent between filters. Using the lukewarm method, the Sterifilt and the cotton filter seem to retain some morphine than the Sterifilt+. With both methods, the wheel filter retains more morphine than the other filters.

### Rinsing of wheel filters

To examine whether more morphine can be recovered by rinsing wheel filters after filtration, these were rinsed using 0.3 mL of extra water. For the cold preparation, rinsing resulted in a slightly higher morphine content (57% versus 51% without rinsing). For the lukewarm technique, the total quantity of morphine recovered was not significantly higher after rinsing (87% instead of 86%).

### Contact and stirring time

The influence of the stirring and contact time between water and the active compound on morphine extraction was tested by stirring for only 20 s. For both preparation methods and for both filters used (Sterifilt+ and wheel filter), the quantity of morphine extracted dropped dramatically by 28 to 49% compared to a stirring time of 1 min.

Stirring duration is thus an important factor. It should be of about 1 min for the lukewarm method. For the cold preparation method, even after 1 min, only a fraction of the morphine was extracted. An enhanced duration might improve extraction in cold water, but will be less acceptable to PWID.

### Preparation of 200-mg capsules

There is no significant difference between the lukewarm extractions of a 100-mg and a 200-mg capsule in 2 mL of water. When using a Sterifilt+, for both formulae, between 93 and 99% of the morphine can be extracted when stirring for 1 min and between 71 and 73% can be extracted when stirring for 20 s.

## Discussion

When dissolving slow release granules of 100 mg morphine sulphate in 2 mL of water, using a cold preparation technique, we harvested on average 29 to 56% of the available morphine sulphate, depending on the contact time. This finding is equivalent to the findings of Cabeças et al. (year unknown) [25] who collected an average of 46% using this method with an unknown contact time.

The hot extraction performed by Cabeças et al. (year unknown) [25] yielded just slightly more morphine (55.3%). Our lukewarm method, on the other hand, yields up to 99% of the morphine. This difference is probably due to a difficulty in recovering all the viscous suspension from the cooker after a hot preparation technique (e.g. after filtration, some of the jelly substance is still in the cooker).

Using this lukewarm method, the extraction of 200 mg of morphine sulphate in 2 mL of water was as efficient as the extraction of 100 mg. When this preparation technique is used, it can be supposed that 100 mg of morphine dissolves in 1 mL, enabling PWID to use low dead space syringes (syringes with an attached needle) for morphine sulphate injections, reducing the risk of viral transmission related to syringe sharing [26, 27].

As mentioned, the virtually complete extraction by Mclean et al. [22], using both a hot and a cold technique, is probably due to a high initial volume (they used 3 mL), a relatively low morphine dosage (60 mg) and a long contact time (5 min). They also experienced quite some loss inside wheel filters (about 36% of the initial 3-mL solution), but a rinse with 1 mL was successful to obtain most of the initial drug. In our study, a rinse with 0.3 mL of water was not able to do so.

We believe that the lukewarm method has some major advantages that make it more readily acceptable to PWID, namely, the use of a low initial volume, the possibility to use small syringes (often associated to the use of smaller needles) or even low dead space syringes, the absence of the obliged rinse to obtain the total quantity of the drug, the need to wait for only 1 min for the drug to be dissolved and the ease and efficiency of filtration following this preparation method.

Regarding particle reduction, the lukewarm preparation practice followed by filtration through a Sterifilt, a Sterifilt+ or a wheel filter has been evaluated as the best preparation practice for slow release morphine sulphate. Among these membrane filters, the Sterifilt+ has the best result regarding filtration time and morphine yield. As with many “best practices”, this method has not been developed by us, but by people who use and inject this drug in the field.

People who use drugs are resourceful. The reason for their reluctance to use a cold water preparation method has been found, and they were proven right. This method does yield less morphine. To spread information and to convince others to use a particular preparation technique, lab tests can help. For PWID, it is not easy to try to change a preparation practice for a valuable drug that they cannot miss and do not want to lose, even partially, even once.

Evidence-based information on solubility and filtration effectiveness can help people to take just that single step that makes their life easier and their drug less harmful in the long run.

## Conclusions

When slow release morphine sulphate capsules are diverted by injection, the use of filters is essential to prevent long term complications due to the injection of insoluble drug fillers such as talc and starch.

This research provided us with a “best preparation practice” for slow release morphine sulphate capsules. This practice consists of preheating water, followed by adding beforehand crushed microbeads to the solution, then waiting and stirring for about a minute, followed by filtration through a membrane filter. The three membrane filters tested were equivalent in number of particle reduction. Filtration ease and time was acceptable for all and best for the Sterifilt and the Sterifilt+. As for active compound yield, though all membrane filters recovered more than 86% of the available morphine, the Sterifilt+ was associated with the highest yield (99%).

The provision of a method that is easy to use, reduces harms associated to the injection of insoluble particles and recovers virtually all the active drug has a large chance to be adopted by people who use drugs. This type of “best practices” can be provided by drug workers and by people who use drugs to actively promote harm reduction.

## Data Availability

Additional data and figures that support the findings of this study are available. Restrictions apply to the availability of these data, which were used under license for the current study, and so are not publicly available. Data are however available from the authors upon reasonable request and with permission of both Ethypharm and the Research and Prevention Fund Apothicom “know more, risk less” (FRPA) (hereafter called (FRPA)).
